# Systematic reviews of diagnostic test accuracy for evidence-based diagnostic practice in Africa

**DOI:** 10.4102/ajlm.v1i1.7

**Published:** 2012-01-30

**Authors:** Eleanor A. Ochodo, Mariska M.G. Leeflang

**Affiliations:** 1 Department of Clinical Epidemiology, Biostatistics and Bioinformatics, Academic Medical Center, University of Amsterdam, Amsterdam, The Netherlands

In 2009, a debate started about whether there was enough evidence to change current guidelines, from presumptive malarial treatment of children under 5 years who present with fever, to testing these children before treating them.^1,2^ A major argument against this policy change was the lack of confidence in the performance of the available rapid diagnostic tests to diagnose malaria in children accurately. This debate neatly illustrates one of the many diagnostic dilemmas with which resource-poor countries struggle. It also illustrates the need for more, and better, evidence about diagnostic questions in these countries.

The ultimate question here may be whether we should test before treatment. The optimum study design would be a randomised controlled trial in which one arm is tested before treatment, and the other arm is treated presumptively. Before such a trial will be granted by ethical committees, health-care workers, the public or the patients, however, more knowledge is needed on the accuracy and reliability of the available malaria tests. Only when the accuracy of these tests is high enough will policy makers, ethical committees, health-care workers and patients put their trust in them. Furthermore, it will be possible to make a sensible decision about which test to use in the trials only when the accuracy of the available tests is known.

The accuracy of a diagnostic test depends on its ability to distinguish people with a target condition from people without the specified condition. A target condition can be either a disease or a specific stage of a disease. Accuracy is expressed often as sensitivity (percentage of people carrying the disease, with a positive test result), and specificity (percentage of people who do not carry the disease, with a negative test result). Other measures to express accuracy include the predictive values, likelihood ratios, area under receiver operating characteristic curve, and diagnostic odds ratios. In a study evaluating the accuracy of a test, the results of the test under evaluation are compared to the results of a reference standard. A reference standard is the best available test or method to diagnose a target condition accurately.^3,4,5^

A valid scientific evaluation of the accuracy of tests is required in order to assist health-care providers, researchers, and policy makers in making rational and evidence-based decisions about the use and interpretation of diagnostic tests. A valid scientific evaluation can be carried out by summarising the results of previously published studies systematically. Such a systematic review is a scientific tool that objectively and methodologically identifies, appraises and summarises the existing data from quality primary studies.^6^ It constitutes the highest level of scientific evidence.^7,8^ A systematic review may, or may not, include a meta-analysis, which is a statistical method of summarising the results of primary studies into a single and precise estimate.^6^ The benefits of systematic reviews are substantial. Firstly, they help to summarise a large volume of information into a form that can be read easily and can be used to make decisions. Secondly, systematic reviews establish whether scientific findings are consistent and can be generalised across populations and settings, or whether findings vary significantly according to subgroups. In addition, systematic reviews are of use in identifying the risk of bias in primary studies. The rigorous methods used in systematic reviews limit bias and improve the reliability and accuracy of conclusions. As a result they can assist effectively in guideline development and in translating research into practice.^9,10^

Systematic reviews of diagnostic test accuracy in particular are useful to establish why the accuracy of tests vary. Test accuracy has been shown to vary with factors such as the patient population, spectrum of disease, disease stage, the type of test used, the method of test administration and the expertise of the people who administer the test.^11^ Diagnostic test accuracy reviews are also useful in comparing tests, or combinations of tests, or different diagnostic strategies.^12^

Effective diagnosis in Africa is impeded by limited financial and human capacity, and poor laboratory infrastructure. Poor laboratory infrastructure includes poor assurance mechanisms or a lack of quality assurance mechanisms, a lack of appropriate laboratory reagents and equipment, and logistical challenges in specimen collection, storage and transportation. These factors may lead to numerous variations or inconsistencies in diagnostic results; therefore, even when laboratory services are available, health workers perceive them as unhelpful and unreliable.^13^ These variations and inconsistencies constitute a hindrance to decision making and guideline development. In this setting, therefore, systematic reviews of available studies on the accuracy of diagnostic tests will be useful in evaluating inconsistencies objectively, and in identifying sources of bias and variation. More so, in a situation where resources are limited, both an accurate diagnosis and the choices made towards that diagnosis are crucial for the optimal use of those resources. The adoption of quality systematic reviews of diagnostic test accuracy will facilitate rational decisions related to which diagnostic tests resources should be allocated.

Unfortunately, the capacity to prepare systematic reviews in developing countries is limited by a shortage of skills and a lack of access to scientific literature because of insufficient finances to pay for subscriptions to medical journals.^14^ Access to scientific literature is hampered further by slow Internet connections that make it difficult to download articles.^15^ Concerted efforts are therefore required to promote the use of scientific literature and to build Internet capacity. In its bid to enhance the quality of laboratory practice in Africa, the newly established African Society for Laboratory Medicine can help to advocate for the use of high standard diagnostic test accuracy reviews to guide evidence-based diagnostic practice.^16^

Currently, the most prominent organisation that is actively promoting the preparation and use of systematic reviews is the Cochrane Collaboration.^17^ Although it is committed to global participation in the use of systematic reviews, the Cochrane Collaboration is still dominated by authors and articles from developed nations. The evidence from Cochrane reviews, therefore, may not be applicable to developing countries, which have the largest burden of disease.^18^

In order to address this challenge, the Cochrane Collaboration has embarked on initiatives to promote participation from developing countries actively. These include building capacity of local authors through the provision of training opportunities, fellowships and by forming partnerships with local institutions. For instance, the Reviews for Africa Programme (RAP) trains and mentors African health researchers to prepare Cochrane reviews. RAP is a partnership between the South African Cochrane Centre and the Liverpool School of Tropical Medicine. This programme focuses on systematic reviews on diseases that are applicable to Africa, such as HIV and AIDS, malaria and tuberculosis.^19^

The Collaboration for Evidence Based Health Care in Africa (CEBHA) was formed recently to further promote evidence-based practice in Africa. This organisation is the result of collaboration between international and local African researchers, with its main aim being the facilitation of knowledge and the implementation of evidence-based health care in Africa. One of its pillars is the support of African scientists to develop systematic reviews through training and mentorship. It is currently focusing on eastern African countries and plans to expand to other African countries in the future.^20^

Additionally, in order to enable scientists from developing countries to gain access to scientific literature, the World Health Organization (WHO), in collaboration with major publishers, set up the Health Internetwork Access to Research Initiative (HINARI). This programme offers free or low cost access to medical journals for eligible developing countries. This enhanced access to literature can enable African researchers to prepare systematic reviews.^21^

Even though Internet connectivity in Africa is still limited, it has witnessed a marked improvement in penetration and speed. Latest estimates report an increase of 2633% in Internet users between 2000 and 2011. Broadband initiatives have also been put in place to increase bandwidth, with the latest being the availability of fibre-optic cables in certain African countries. As Internet access and speed continue to improve, access to scientific literature will improve as well.^22^

In a nutshell, systematic reviews, when prepared rigorously, objectively summarise the findings from available studies and provide a strong source of evidence. In order to promote the use of diagnostic test accuracy reviews in Africa, the African Society for Laboratory Medicine can help to promote the use of high standard diagnostic test accuracy reviews to guide evidence-based diagnostic practice. Secondly, the importance of these reviews ought to be disseminated to African researchers through workshops, and their capacity needs to be built through training and mentorship. These can be performed in partnership with the Cochrane Collaboration, CEBHA, or other researchers well versed in developing these reviews. Finally, the use of the HINARI programme that was set up to enable researchers from developing countries to access scientific literature should be encouraged.
